# A mixed-methods investigation of an ecological momentary assessment protocol for cigarette-smoking youth: Psychometric properties and participant experiences

**DOI:** 10.1016/j.dadr.2024.100314

**Published:** 2024-12-25

**Authors:** Suhaavi Kochhar, Hanneke Scholten, Dominique F Maciejewski, Michelle A Pingel, Maartje Luijten

**Affiliations:** a Radboud University, Postbus 9102, Nijmegen 6500 HC, the Netherlands; b Erasmus Medical Centre, PO Box 2040, Rotterdam 3000 CA, the Netherlands; c Tilburg University, PO Box 90153, Tilburg 5000 LE, the Netherlands

**Keywords:** Ecological momentary assessment, Middle adolescence, Late adolescence, Cigarette, Smoking, Psychometric properties, User experience, Mixed-methods

## Abstract

**Introduction:**

Ecological momentary assessment (EMA) is popular in smoking research to study time-varying processes and design just-in-time personalised cessation interventions. Yet, research examining the psychometric properties of EMA and user experiences with EMA protocols is lacking. We conducted a mixed-methods study to test the EMA component of a mobile intervention for middle to late-aged adolescents (16–20 years) who smoke cigarettes at least weekly.

**Method:**

Participants (*N* = 84) filled out global self-report measures at pre- and post-test, and reported their craving, mood, and smoking behaviour five times daily for seven days via their phones. We tested intra-class correlations, convergent validity, test-retest reliability, and multilevel internal consistency of the EMA items. Further, participants answered qualitative questions about their experiences with the EMA including the timing and frequency of assessments, clarity of individual items, and how it impacted their daily lives.

**Results:**

The EMA questionnaires showed good convergent validity and reliability. The average compliance rate was 77 %, and generally, participants evaluated the experience positively. While most participants rated the timing and frequency of EMA positively, some participants did not like that assessments expired after 1.5 h. Forty percent of participants also reported that they liked monitoring their smoking and mood.

**Conclusions:**

The use of EMA in our target group is feasible and had good compliance. The items used are suitable for researchers to use in future studies. We urge researchers to test the psychometric quality and feasibility of new EMA protocols before using them in confirmatory research.

## Highlights

• The EMA items showed test re-test reliability and convergent validity.
• Craving and affect vary momentarily, warranting intensive longitudinal measurement.
• EMA is feasible among smoking youth with good compliance (77 %)
• Positive participant experiences included self-reflection on smoking behaviour.
• Negative participant experiences included EMA expiration and repetitive questions.

Ecological momentary assessment (EMA) is a method of collecting data through intensive repeated measurements in daily life and is a suitable method for studying smoking and related constructs (Shiffman, 2009, Shiffman et al., 2008, Stone and Shiffman, 1994). EMA provides greater ecological validity and lower recall bias than global self-report measures (Shiffman, 2009, Shiffman et al., 1997, Wright and Zimmermann, 2019). Yet, the psychometric properties of EMA questionnaires are often not tested. EMA studies also put an additional burden (Eisele et al., 2022) on participants which might be experienced differently across target groups. In the present study, we used a mixed-methods approach to a) test the psychometric quality of the EMA measures and b) study how cigarette-smoking youth (middle and late-aged adolescents; 16–20 years) experienced the EMA protocol.

EMA research in smoking is centred around two aims. First, processes surrounding smoking and cessation known to fluctuate momentarily, such as craving and affect, are examined (Eisele et al., 2022, Hedeker et al., 2009, Shiffman et al., 2007) to obtain a more detailed, time-specific understanding of these dynamic, complex processes. Second, EMA is used in personalized smoking cessation interventions, by, for example, using real-time input of affective and/or behavioural data. The data in ecological momentary interventions (EMIs) are used to send tailored, in-the-moment messages at moments when the odds of smoking lapse are higher (Abroms et al., 2017, Hébert et al., 2020, Ruscio et al., 2016, Abroms et al., 2014, Krishnan et al., 2019, Mason et al., 2015, Mason et al., 2016). A recent meta-analysis revealed that participants who received EMIs reported greater abstinence rates than control groups (Eghdami et al., 2023). However, EMIs naturally depend on the psychometric quality and feasibility of EMA protocols to ensure accuracy and usability.

Although the use of EMA in smoking research is becoming increasingly popular and shows promise, methodological concerns remain. First, the psychometric properties of EMA items are often unknown (Cloos et al., 2023, Eisele et al., 2021, Shiffman, 2009, Wright and Zimmermann, 2019, Kuppens et al., 2022). For example, many EMA studies borrow items from existing global measures of smoking-related outcomes, like craving or withdrawal symptoms, which were originally designed for longer timeframes (e.g., past week, past month). While these items are often adapted, such as changing the wording to fit the intensive longitudinal context, there are many processes involved in responding to EMA that researchers cannot estimate, such as item interpretation by participants and the relevance of measuring a construct daily. These processes can also influence the validity and reliability of the EMA. For instance, we cannot safely assume that items on withdrawal symptoms experienced over the last week are sensitive to moment-to-moment changes in withdrawal symptoms when administered multiple times a day. Thus, we should test the reliability and validity of such items in an EMA context specifically.

EMA methodologists have proposed several approaches to test the psychometric quality of EMA items in the relevant target group, such as testing the convergent validity, test re-test reliability, and multi-level intra-class correlations. Participants’ perceptions and interpretations of questions and experience with the sampling schedule also provide insight into the validity of the EMA measures. A useful approach to gain such insights is to conduct exploratory (pilot) tests of the EMA protocol, which maintains focus on the user (Dejonckheere and Erbaş, 2021, Eisele et al., 2021).

Pilot-testing EMA designs also addresses a second limitation, which is the lack of participatory research on EMA designs. Studying response processes is an important yet neglected source of validity evidence (American Educational Research Association et al., 2014). Items only quantitatively tested for psychometric properties may not tell us whether participants understood items as we intended or if they were able to answer them. Using participatory research researchers can design new EMA items through focus groups with participants to learn which items are most salient to their (daily) experiences (Soyster and Fisher, 2019) and what phrasing gets the message across best. Alternatively, researchers can ask qualitative questions at the end of a (pilot) study to know how participants interpreted items and experienced the EMA sampling approach. These qualitative insights can teach us more about the feasibility of EMA designs and psychometric properties of EMA items directly from participants. Overall, mixed-method tests of an EMA design can provide insight into design flaws, feasibility, and psychometric quality.

Through the present pre-registered study (osf.io/7zcps/), we use mixed methods to study the psychometric properties and participatory experiences of an EMA protocol designed for a smoking cessation game intervention that we are developing for smoking youth. We test the convergent validity, reliability, usability, and feasibility of our EMA protocol using quantitative and qualitative approaches. Our method and findings are relevant to fellow researchers, because 1) we conduct the study in a sample of ad libitum smoking youth (16–20 years), providing insights for research in such samples, 2) we provide insight into the psychometric properties of items previously used in smoking (EMA) research, but not previously tested for psychometric quality, and 3) we illustrate the importance of qualitative research while studying participants’ perceptions and experiences with the EMA protocol to better implement it in our intervention.

## 1 Method

### 1.1 Participants

Sample size was based on feasibility, i.e., time and funding, in the context of a larger study. We used convenience sampling through social media advertisements and educational institutions. Inclusion criteria were 1) smoking cigarettes at least weekly, 2) being 16–20 years old, and 3) being (slightly) motivated to quit smoking cigarettes. The sample included 84 smoking youth (*M*_age_ = 17.7; *SD*_age_ = 1.5; Supplementary Materials *I*), of whom 58 % were female.

### 1.2 Procedure

Interested participants filled a screening questionnaire assessing the inclusion criteria. Eligible participants received a pre-EMA questionnaire measuring demographics, nicotine dependence, smoking urge, nicotine withdrawal questions, and instructions to download the EMA mobile application (Avicenna, 2024). Participants completed EMA five times daily for seven days at a random moment within the intervals 09:00–09:30, 12:00–12:30, 15:00–15:30, 18:00–18:30, and 21:00–21:30. The surveys were available for 1.5 h from the time they were triggered with a reminder 15 min before it expired. At the end of the EMA, they received a post-EMA questionnaire assessing nicotine withdrawal, positive and negative affect, and qualitative questions about the EMA.

The study was conducted entirely in Dutch and ethical approval was obtained from the University of Twente in the Netherlands (application number 221060). Participants were reimbursed €0.50 per EMA and €2.50 for the pre- and post-EMA questionnaires together. Thus, they could earn up to €20 or equivalent study credits for participating.

## 2 Measures

### 2.1 Ecological momentary assessments (EMA)

#### 2.1.1 Affect

We measured affect using items from the ESM repository (Kirtley et al., 2018). We measured positive affect using one high arousal item, “joyful”, and one low arousal item, “relaxed”. Similarly, negative affect included “restless” as high arousal and “sad” (Kirtley et al., 2021) as low arousal. Participants reported how they felt “right now” on a VAS scale from 0 (not at all) to 100 (extremely).

#### 2.1.2 Smoking withdrawal

We used three single items chosen for the 6-item short-form of the Wisconsin Smoking Withdrawal Scale (WSWS2; Smith et al., 2021). Participants rated being bothered by “wanting to smoke” and “having trouble concentrating” in the moment, and “troubled sleep” the previous night on a VAS scale from 0 (not at all) to 100 (extremely). The sleep item was only asked in the first EMA of each day.

#### 2.1.3 Smoking

Our aim was to measure participants’ smoking patterns and estimate daily use. We modified an item from the ESM repository, “How many cigarettes have you smoked?” (Bennik et al., 2015). In the first EMA each day, we asked (1) whether they smoked the last evening after they answered the last EMA and (2) if they smoked since they woke up. In all the other EMAs of the day, we asked (3) if they smoked since they last answered an EMA. The items remained the same also if participants missed an assessment. If participants reported smoking, they were asked how many cigarettes they smoked.

### 2.2 Global retrospective measures

We also used multiple static self-report measures at pre- and/or post-test to test the convergent validity of the EMA items (Supplementary Materials *II*). Affect was measured at post-test using the Dutch version of the 20-item Positive and Negative Affect Schedule (Peeters et al., 2004). We used the Dutch version of the Fagerström test for Nicotine Dependence (FTND; Vink et al., 2005) to measure nicotine dependence at pre-test. Urge to smoke was assessed using the Dutch version (Littel et al., 2011) of the Questionnaire of Smoking Urges (Cox et al., 2001, Tiffany and Drobes, 1991) and a single-item measure as used in our previous studies (Scholten et al., 2019). The 19-item WSWS2 (Smith et al., 2021) was used to measure withdrawal symptoms at pre-EMA and post-EMA. As in our previous studies, we measured motivation to quit smoking using a single-item measure (Scholten et al., 2019) at screening, pre-EMA, and post-EMA. Finally, participants reported how many days in a week they typically smoked and how many cigarettes they smoked in a day that they smoked. The days and cigarettes per day were then multiplied to assess weekly smoking at pre-test.

To assess participatory experiences, we asked participants about different aspects of the EMA including the schedule and frequency, clarity of items, and measurement reactivity. We included questions such as “How did you feel about the number of questionnaires in a day?” and “Were any of these items unclear to you? Please elaborate.” (All EMA and retrospective measures can be found at osf.io/7zcps/).

## 3 Data analysis strategy

All analyses were conducted in R v3.2.1 (R Core Team, 2023). First, we calculated intra-class correlations (ICC) in multilevel models per EMA variable (Eisele et al., 2021) to assess whether item responses varied sufficiently within-person to warrant frequent momentary measurement (i.e., ICC between 0.20 and 0.40; Bolger and Laurenceau, 2013). Next, we plotted each participant’s data per EMA variable to visually explore the variance over time. Third, we split the EMA data per variable into odd and even measurement days and calculated the correlation between them as a measure of test re-test reliability (Wendt et al., 2020, Mejía et al., 2014). Fourth, we calculated multilevel internal consistency (Nezlek, 2017) of positive and negative affect items. Fifth, we measured the convergent validity by calculating correlations between the mean EMA score to the corresponding score on a pre- or post-EMA measure (Forkmann et al., 2018). We also explored the missingness in EMA data for each day and each assessment separately to know how representative the data is of the intended time samples.

Finally, we explored descriptive statistics for quantitative items on user experience and performed thematic content analysis for qualitative questions on user experience (Braun and Clarke, 2006). As part of the qualitative data analysis, two independent raters inductively analyzed responses to a unique set of questions each. The raters first developed codes as they independently read the data (open coding). Next, they looked for similarities in codes clubbed similar codes and teased apart complex codes (axial coding) in their respective data. Finally, the independent rates rated 10 % of data from their fellow rater using the existing coding scheme to test inter-rater reliability. We discussed coding discrepancies internally. Inter-rater reliability was excellent (ICC = 0.95; Supplementary Materials IV).

## 4 Results

### 4.1 Descriptive statistics

On average, participants reported smoking 54 cigarettes weekly (*SD* = 41.70; Range: 3–200) and a moderate motivation to quit (*M* = 2.87, *SD* = 1.14, Range: 1–5). Participants completed on average 27 out of 35 EMA, indicating good compliance (76.89 %, *SD* = 5.81, Range: 6–35).

### 4.2 Psychometric properties of EMA

**Fig. 1:**
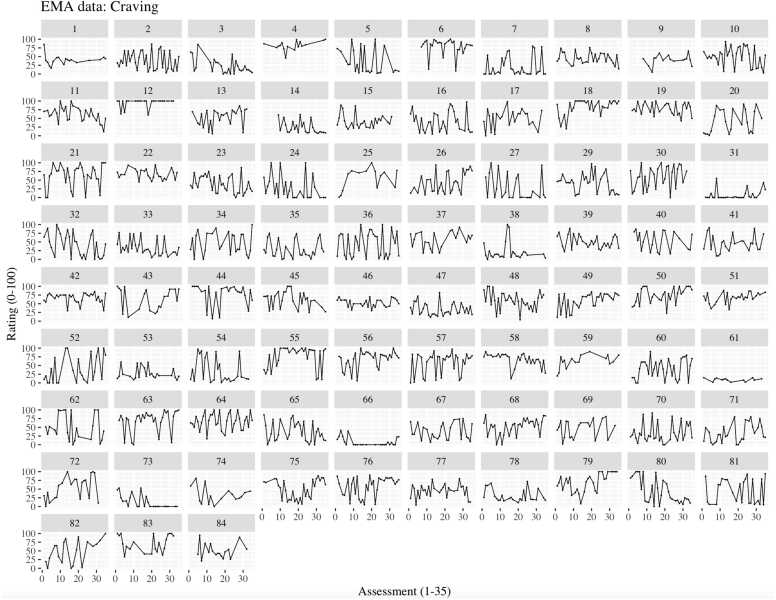
Craving ratings per EMA per participant. Note. Each square plot represents a unique participant’s ratings for momentary craving (Y-axis) in up to 35 assessments (X-axis). Within the plots, each plotted point represents a measurement.

Multilevel ICCs (Table 1) suggested noteworthy variance in each item at the within-person level. Simple plots per EMA variable across measurements also showed sufficient within-person variance (see Fig. 1 for craving and Supplementary Materials *III* for all EMA items).

**Table 1:** Multilevel intra-class correlations (ICC) and Pearson's correlations between mean scores on odd and even days per EMA item.

EMA item	ICC	Correlation odd and even days	95 % CI	
			*LL*	*UL*
Joyful	.29	.86	.79	.91
Relaxed	.20	.80	.71	.87
Restless	.28	.80	.71	.87
Sad	.29	.80	.71	.87
Craving	.33	.86	.71	.87
Difficulty concentrating	.25	.82	.74	.88
Troubled sleep	.33	.48	.29	.63
Daily smoking	.39	.82	.73	.88

Note. All correlations are significant at p < 0.001

Mean EMA scores on odd days and even days had strong positive correlations, indicating high re-test reliability (Table 1). Multilevel internal consistency analysis revealed that positive affect had a between-person reliability of.93, 95 % CI [.89,.97] and a within-person reliability of.65, 95 % CI [.62,.68]. Negative affect had a between-person reliability of.87, 95 % CI [.81,.94] and a within-person reliability of.58, 95 % CI [.55,.62]. These results suggest that the reliability between participants was high. However, within participants, the responses were not as reliable implying that individual participants’ ratings of positive and negative affect varied more within individuals.

All mean EMA scores were positively correlated with corresponding global self-report measures at pre-test or post-test (Table 2).

**Table 2:** Convergent validity: Pearson's correlations between mean scores on EMA variables and corresponding global measures at pre- or post-test.

EMA variable	Global scale (subscale)	Pearson's correlation	95 % CI	
			*LL*	*UL*
Positive affect	PANAS (positive)	.31	.22	.58
Negative affect	PANAS (negative)	.55	.40	.70
	WSWS (negative affect)	.53	.35	67
Craving	WSWS (craving)	.67	.53	.78
	QSU	.45	.27	.61
	Craving single-item	.58	.42	.71
Concentration	WSWS2 (concentration)	.61	.45	.73
Sleep	WSWS2 (sleep)	.64	.49	.75
Smoking	FTND	.43	.24	.60
	Weekly smoking (WS-A)	.50	.32	.64
	Weekly smoking (WS-B)	.31	.10	.49

Note. EMA = Ecological Momentary Assessment; PANAS = Positive and Negative Affect Schedule; WSWS = Wisconsin Smoking Withdrawal Scale- Revised; QSU = Questionnaire of Smoking Urges; FTND = Fagerström test for Nicotine Dependence; All correlations are significant at p < 0.001 except WS-B which is significant at p < 0.01.

### 4.3 User experience and feasibility of EMA

First, participants rated the overall experience positively on a scale from 1 to 7 (*M* = 5.41, *SD* = 1.13, Range: 2–7). Thirty-one participants (38.8 %) liked gaining insight into their feelings and behaviour. Seventeen participants (21.3 %) did not like answering the same questions every time, while sixteen (20 %) enjoyed monitoring their behaviour. These numbers are not mutually exclusive. Further, participants rated the timing and frequency of the EMA positively (*M* = 4.46, *SD* = 1.53, Range: 1–7). Fifty-four participants liked the number of questions (up to 11 items; 67.5 %) and forty-three were satisfied with the timings (53.8 %). Some reported they were often busy when the EMA were sent (28.8 %), or found the questions to be too many (27.5 %). We also asked participants to point out items that were unclear or difficult to answer. The majority thought all questions were clear (76.3 %) or easy to answer (72.5 %). Five (6.3 %) participants found it difficult to rate restlessness because they could not separate being busy from being restless or did not clearly understand the meaning. Five (6.3 %) participants found the smoking question unclear in the time point it referred to with “last answer”. Four (5 %) participants found it difficult to report “difficulty concentrating” if they were not actively concentrating on something at that moment.

Participants self-reported measurement reactivity (“Was there an effect of the questionnaire on your daily life?”) on a scale from 1 (not at all) to 7 (very much; *M* = 3.17, *SD* = 1.58, Range: 1–6). Thirty-seven (46.3 %) said they gained insight into their own smoking behaviour and/or feelings. Twenty-three (28.8 %) reported perceiving no effect. Twelve (15 %) participants thought they smoked less during the study. Finally, we asked participants what they would do differently if they were the researchers. About half of the participants (53.8 %) said they would not change anything. Twenty-two (27.5 %) participants suggested new/alternate questions (all qualitative results are in Supplementary Materials VI).

### 4.4 Exploratory analyses

First, since participants rated measurement reactivity as moderate, we conducted exploratory analyses on changes in smoking over the days of EMA. We tested the relationship between daily smoking and day of EMA (1−7) in the whole sample and a subset of participants who reported experiencing moderate measurement reactivity (*n* = 48) using multilevel models with data nested in participants. Day of EMA did not predict daily smoking in both samples (Supplementary Materials VII*)*.

The correlation between the retrospective positive affect scores and mean EMA scores on positive affect was lower than expected. Therefore, we also tested how each EMA positive affect item correlated with retrospective positive affect scores. Mean scores on the “joyful” item correlated moderately with retrospective positive affect (*r* = .46, *p* < .001) and mean scores on the “relaxed” item correlated weakly with retrospective positive affect (*r* = .34, *p* = .002).

**Fig. 2:**
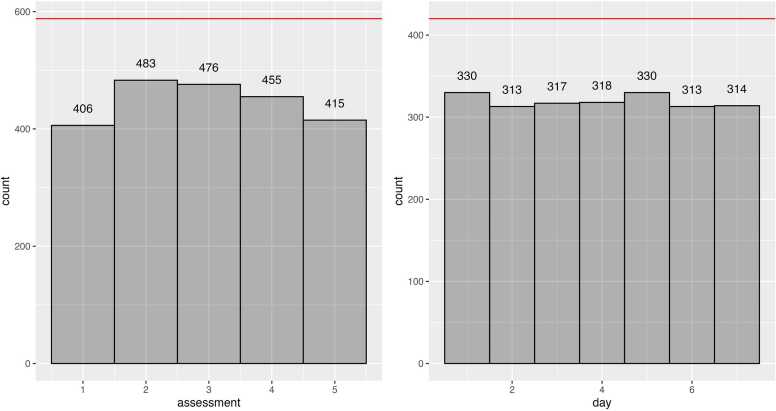
Sample-level missingness per assessment and per day of EMA. Note. Participants (N = 84) filled EMA five times daily for seven days. Red lines indicate the maximum number of responses in the dataset per assessment moment and day of assessment.

We also plotted missingness data per day (days 1–7 of EMA) and per assessment (assessments 1–5) across the sample (Fig. 2) and per participant (Supplementary Materials V). Missingness did not seem to vary based on assessment time or day. Further, we tested the relationship between demographic variables such as age, gender, weekly smoking, and education level, and EMA compliance and found no significant relations (Supplementary Materials VII).

## 5 Discussion

The aim of the current study was to test the psychometric properties and participant experiences of our EMA protocol measuring smoking, mood, and withdrawal in a sample of smoking youth. All EMA items showed substantial variance, good convergent validity, and good test-retest reliability. We also studied participants’ experiences with the EMA to understand the feasibility of the protocol. Compliance was generally high, and participants rated participation positively. Yet, some participants did not like answering the same questions repeatedly or that the EMA expired after some time. Although some participants reported measurement reactivity, the smoking data did not reflect changes in smoking over the week of EMA.

### 5.1 Psychometric insights

Overall, the EMA showed good psychometric properties. All EMA items had ICCs indicating sufficient within-person variation (Bolger and Laurenceau, 2013). Further, simple visualisations of EMA data per participant over time show noteworthy fluctuations (Fig. 1). We can infer that the EMA items were sensitive to momentary changes in affect, craving, and difficulty concentrating and warrant the use of intensive repeated measurements for these constructs.

EMA data from odd and even days were strongly correlated with each other indicating that the fluctuations were not random. Notably, the correlation between troubled sleep ratings on odd and even days was only moderate. We can attribute this to the once daily measurement of sleep variable, resulting in fewer data points. Thus, psychometric tests of items measured only once a day warrant more than 7 data points.

We found reasonable evidence for convergent validity through comparisons of mean EMA scores and global retrospective measures. The moderate effect sizes are acceptable and as expected (Forkmann et al., 2018, Abma et al., 2016). However, the mean scores from the positive affect EMA items, chosen from the ESM repository, and the positive affect score from the PANAS had a lower correlation coefficient of.31. We can argue that the PANAS covers a wider, yet slightly different, range of 10 positive emotions (such as “interested”, “strong”, “inspired”) while the EMA items referred specifically to feeling “joyful” (high arousal) and “relaxed” (low arousal). On the surface, the closest item from the positive affect subscale of the PANAS to “joyful” is “excitement” and there is no item resembling “relaxation”. This discrepancy was confirmed by exploratory analyses that showed a weaker correlation between the “relaxed” item and the PANAS score than the “joyful” item. Thus, the two scores may not represent the same underlying construct and it might be more valid to study the ”joyful” item as a stand-alone measure of positive affect in future studies.

Some participants reported measurement reactivity, such as smoking less during the study or becoming more aware of their mood. However, exploratory analysis revealed no significant change in daily smoking over the EMA period. Thus, we do not expect that the EMA induced changes in smoking behaviour over the 7 days of EMA, which is in line with a previous study (Rowan et al., 2007). Although we did not find evidence of reactivity in smoking behaviour, there could have been reactivity while reporting mood, craving, or difficulty concentrating as reported in some previous research (Eisele et al., 2023, Rowan et al., 2007, Wrzus and Neubauer, 2023). To improve the validity of EMA data, it is important to be aware of biases that may be introduced by answering the EMA. We urge researchers to account for reactivity when designing EMA protocols to both, test dynamic relationships between psychological and behavioural constructs, and while designing EMA-based interventions.

Further, we inferred that the EMA items tested in our study are suitable for use in future smoking research in youth. We chose these items from existing global measures and the ESM repository with slight modifications for our design. The items and corresponding data are openly available for use (https://osf.io/7zcps/). Further, we recommend that researchers designing EMA protocols first consult the ESM repository (Kirtley et al., 2018). Re-using these items would guide independent researchers designing EMA protocols and support psychometrics by adding synchrony across EMAs.

### 5.2 Participatory insights

We also studied participants’ experiences with the EMA. Participants were compliant (76 %), similar to the mean compliance in other EMA studies (Heron et al., 2017, van Roekel et al., 2019, Wrzus and Neubauer, 2023). Further, the EMA data did not show trends in missingness based on which day of the study it was or which assessment of the day it was. Such random missingness suggests that we did not repeatedly miss out on certain events, supporting the validity of the data.

We used some compliance-enhancing approaches, such as rewarding participants with EUR 0.50 for each EMA they did, sending messages when participants did not answer more than 3 of 5 assessments the previous day, and sending reminders 15 min before an EMA expired. Our experience is in line with previous reviews suggesting compliance-based cumulative reimbursements (Beckham et al., 2008), real-time monitoring of responses, and personal contact with participants are among the most promising compliance-enhancing practices.

Finally, during recruitment, we explicitly expressed our interest in learning about participants’ smoking behaviours. We named the study “When do you smoke?” in Dutch and we advertised that participants could contribute to science by sharing their experiences in our study. In fact, many participants indicated that they liked contributing to scientific research and gaining self-insight. Thus, we hypothesize that emphasizing participants’ contribution to science while advertising the study helped. Moving further, EMA studies can also be advertised as providing insight into one’s behaviour. We know of two recent projects (Fried et al., 2023, Maciejewski et al., 2022) where participants received personal reports at the end of the study. Not only do participants gain insight into their own experiences, but they can also provide interpretations of their EMA data at the end of the study, allowing us to substantiate our quantitative data with qualitative insights.

### 5.3 Limitations

First, our target sample size of 80 participants was based on feasibility, rather than a power calculation. Yet, since our study was designed to be an exploratory test of an EMA protocol with a focus on reliability and feasibility, performing an a-priori power analysis was not warranted. Second, most items used in our EMA protocol were single-item measures. Our items were based on the need within the smoking cessation intervention we are designing, and we wanted to keep the participant burden as low as possible. However, methods to measure the validity and reliability of single items are limited. Yet, EMA researchers are working on psychometric tests for single items (Dejonckheere et al., 2022), such as measuring test-retest reliability by administering one EMA item twice in each assessment.

### 5.4 Recommendations and conclusion

Through the present study, we tested the reliability and feasibility of EMA in smoking youth, in the context of our smoking cessation intervention. Our protocol was feasible, demonstrated acceptable psychometric quality, and is available for use in future studies. Yet, the feedback from participants helped us adjust the protocol before implementing it in our smoking cessation mobile application. For example, few participants found the smoking question in the EMA (“Have you smoked a cigarette since you last answered?”) unclear regarding what time point it referred to. To improve this, we added a visual element in our app that shows users which EMA of the day they are filling in and how many they filled in before.

Based on our experience, we urge researchers to (pilot) test the validity, reliability, and feasibility of EMA protocols before testing hypotheses and/or including an EMA component in an intervention (Dejonckheere and Erbaş, 2021, Eisele et al., 2021). In addition to the methods presented in the paper, researchers can also test discriminant or divergent validity by testing correlations with retrospective measures of contrasting variables. However, it may not always be feasible to preliminarily test EMA protocols. Alternatively, qualitative questions on participant experiences can be included during a post-test measurement.

In conclusion, EMA is not only a valuable tool to collect data in youth smoking research but can also provide participants with insightful experiences. Existing EMA research on complex dynamics in smoking, cessation, and relapse shows great promise. Moving forward, EMA should be designed using protocols that are psychometrically tested in the target group while also accounting for participants’ experiences. When previously tested protocols are not available, new protocols can be (pilot) tested to examine the psychometric properties of the items and study participant experiences with the protocol, following the example of the current mixed-methods approach.

## Funding

This work was supported by the 10.13039/501100004622KWF Kankerbestrijding (grant number 12946).

## CRediT authorship contribution statement

**Suhaavi Kochhar:** Writing – review & editing, Writing – original draft, Visualization, Validation, Software, Project administration, Methodology, Investigation, Formal analysis, Data curation, Conceptualization. **Maartje Luijten:** Writing – review & editing, Supervision, Resources, Project administration, Methodology, Investigation, Funding acquisition, Conceptualization. **Michelle A Pingel:** Writing – review & editing, Software, Project administration, Methodology, Investigation, Conceptualization. **Dominique F Maciejewski:** Writing – review & editing, Supervision, Methodology, Investigation, Funding acquisition, Conceptualization. **Hanneke Scholten:** Writing – review & editing, Supervision, Resources, Project administration, Methodology, Investigation, Funding acquisition, Conceptualization.

## Declaration of Generative AI and AI-assisted technologies in the writing process

During the preparation of this work the author(s) used ChatGPT in order to shorten few (< 5) paragraphs of the manuscript. After using this tool/service, the author(s) reviewed and edited the content as needed and take(s) full responsibility for the content of the publication.

## Declaration of Competing Interest

The authors do not have any competing interests to declare.

## Footnotes

Appendix A Supplementary data associated with this article can be found in the online version at doi:10.1016/j.dadr.2024.100314.
