# Supplementary material for: A mixed-methods investigation of an ecological momentary assessment protocol for cigarette-smoking youth: Psychometric properties and participant experiences
Source: Drug Alcohol Depend Rep. 2024 Dec 25;14:100314. doi: 10.1016/j.dadr.2024.100314 (PMC11759552; doi:10.1016/j.dadr.2024.100314)
Supplement: Supplementary file 1 — Supplementary material [file mmc1.docx]

Supplementary Materials

1. **Sample description**

We screened 230 individuals through social media ads and educational institutions in the Netherlands of whom 201 were eligible. One hundred and four participants eventually participated, including four older than 20 years who were included in the context of classroom recruitment. Two participants quit the study and 14 were removed on being unresponsive for two or more consecutive days.

Thirty-nine (46%) participants were from universities, 37 (44%) from vocational institutions, and 8 (10%) from applied sciences schools.

Forty-nine participants were female (58%), 31 were male (37%), two were 3rd gender/non-binary (2.5%), and two preferred not to share (2.5%).

Inclusion criteria for motivation to quit smoking was tested using the item: “Indicate the extent to which you currently plan to quit smoking.” Participants who rated more than 1 on a scale of 1 (not at all) – 5 (very much) were included.

1. **Global retrospective measures**

**1. Affect.** Positive and negative affect were measured post-EMA using the Dutch version (Peeters et al., 2004) of the 20-item Positive and Negative Affect Schedule (Watson et al., 1988). Participants reported the extent to which they experienced the listed feelings in the last week (i.e. EMA week). Sum scores of 10 items were calculated for positive and negative affect each. The Cronbach’s α was .79 for positive affect and .87 for negative affect in the present study.

**2. Smoking Urge.** We measured urge to smoke using the Dutch version (Littel et al., 2011) of the Questionnaire of Smoking Urges (Cox et al., 2001; Tiffany and Drobes, 1991) at pre-EMA. Participants rated how much 10 items about craving applied to them at that moment. Responses were added for a sum score. Further, we also measured smoking urge using a single-item measure as used in our previous studies (Scholten et al., 2019). Participants could enter a rating between 0 and 100. We found a high Cronbach’s alpha of .91 in our study.

**3. Nicotine Withdrawal.** We used the 19-item revised Wisconsin Smoking Withdrawal Scale (WSWS2; Smith et al., 2021) to measure withdrawal symptoms at pre-EMA (as in the original scale with reference to the last 24 hours) and post-EMA (in the last week, i.e. the EMA week). A mean score was calculated for the whole scale and per sub-scale – craving, concentration, hunger, sleep, negative affect, and restlessness. To the best of our knowledge, a Dutch version of this scale was not available. Bilingual researchers in our team independently translated the WSWS2 to Dutch and then back translated it to English. We resolved any wording discrepancies through discussions, keeping our target sample in mind. The WSWS2 had good internal consistency both pre- (α = .89) and post-EMA (α = .87) in our study.

**4. Nicotine Dependence.** We used the Dutch version (Vink et al., 2005)of the Fagerström test for Nicotine Dependence (Heatherton et al., 1991). Nicotine dependence was calculated using sum scores and the scale was administered in the pre-EMA questionnaire. We found a Cronbach’s α of 0.71 in the present study.

**5. Weekly Smoking.** We calculated weekly smoking retrospectively at the pre-EMA moment in two ways. First, participants reported how many days in a week they typically smoked and how many cigarettes they smoked on a day that they smoked. The days and cigarettes per day were then multiplied to assess weekly smoking (WS-A). Second, participants answered the same questions separately for days of the working week (Monday through Thursday) and in the weekend (Friday through Sunday), the products of which were summed to obtain the WS-B score. The correlation between WS-A and WS-B in the present study was strong, r = .75, p < .001.

**6. Motivation to Quit.** As in our previous studies (Scholten et al., 2019), we measured motivation to quit smoking using a single-item measure at screening, pre-EMA, and post-EMA. Participants rated how motivated they were to quit smoking at that current moment on a 5-point Likert scale. We included participants at least slightly motivated to quit and continued to measure motivation to quit pre- and post-EMA since we are designing the EMA for use in an intervention for youth who would like to quit smoking.

1. **EMA data plots**


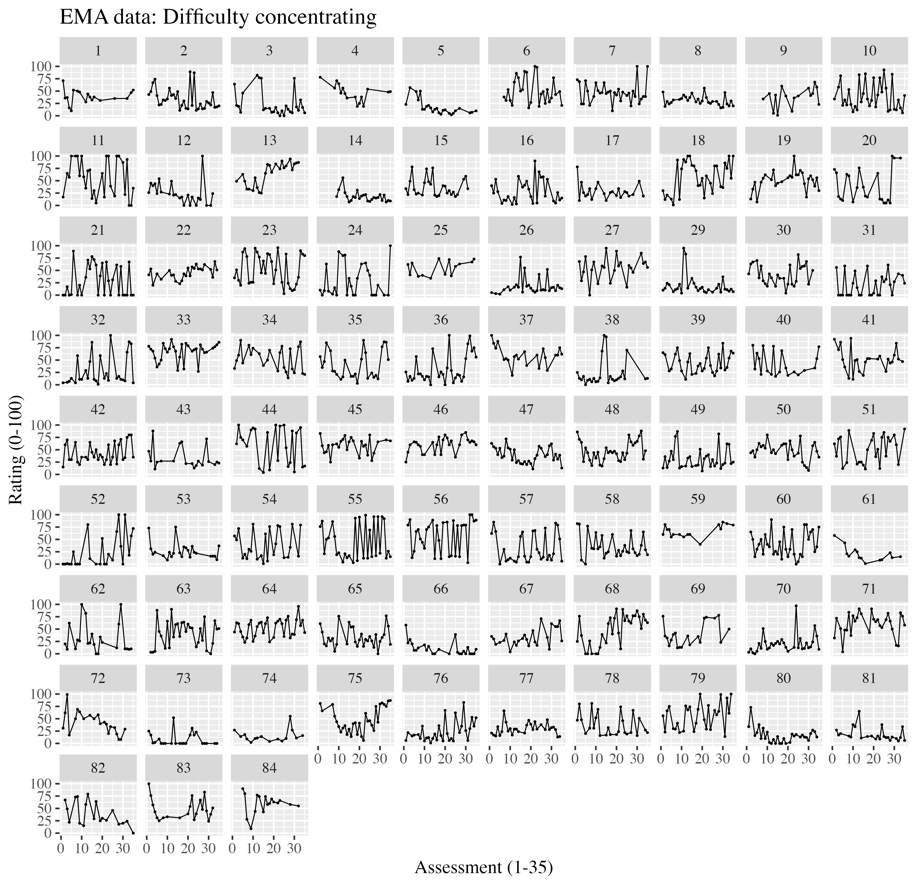

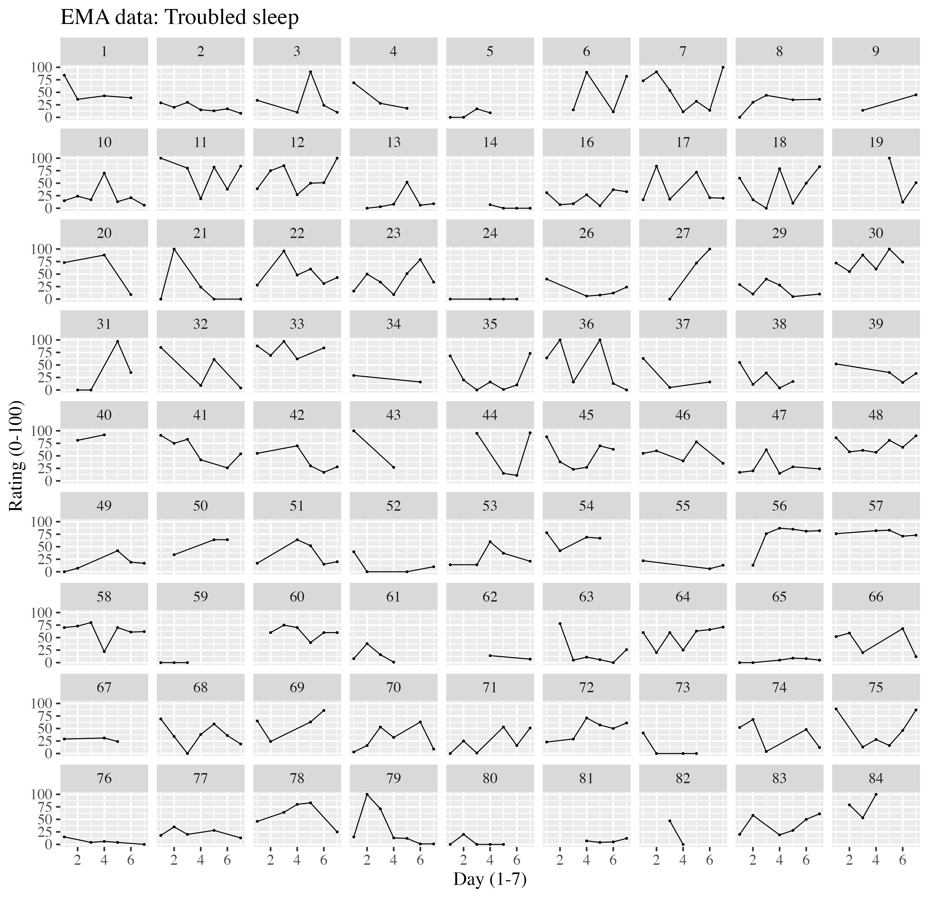


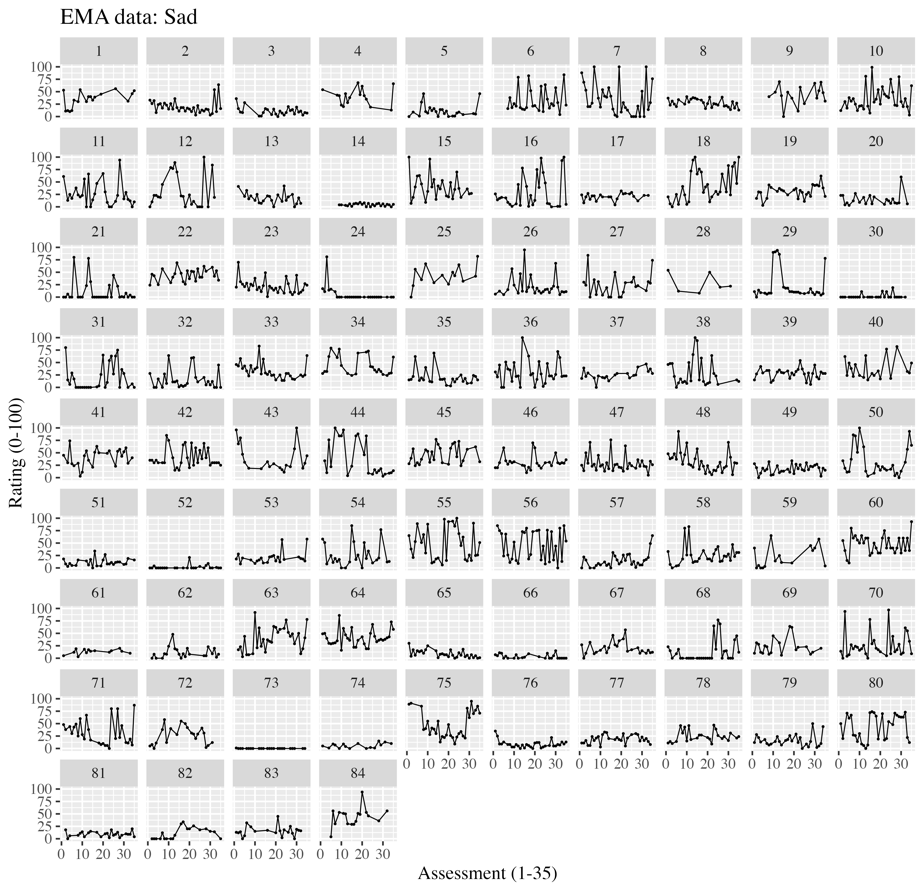

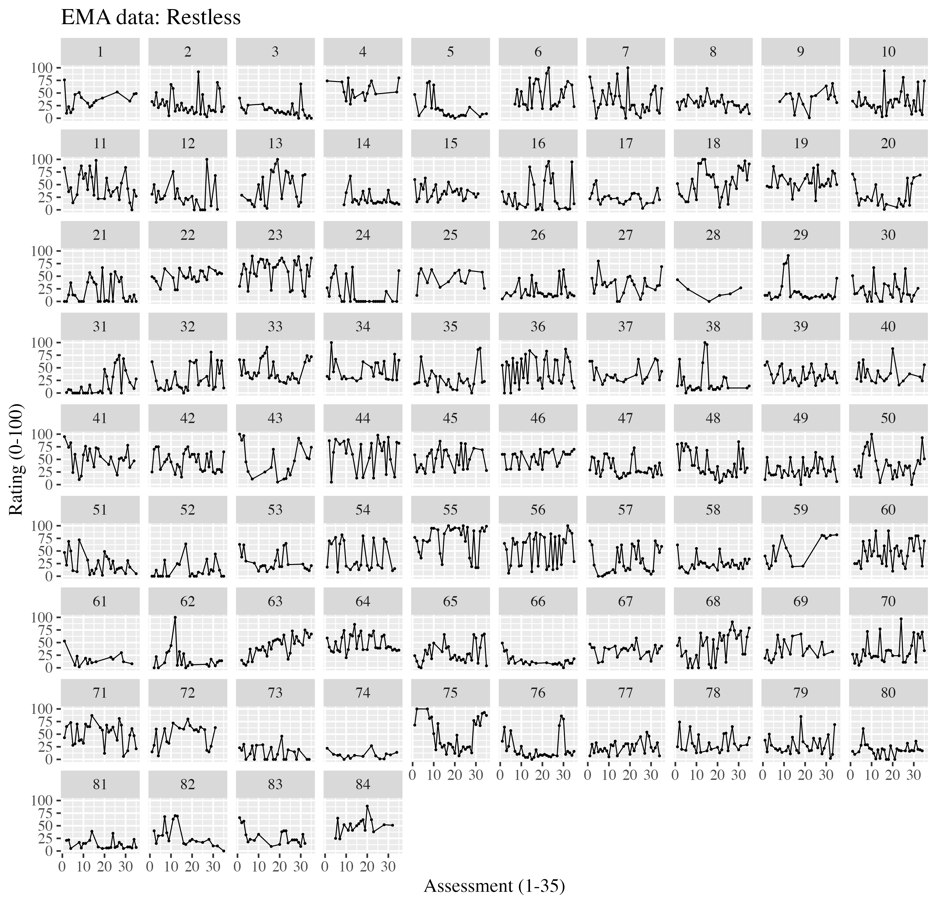


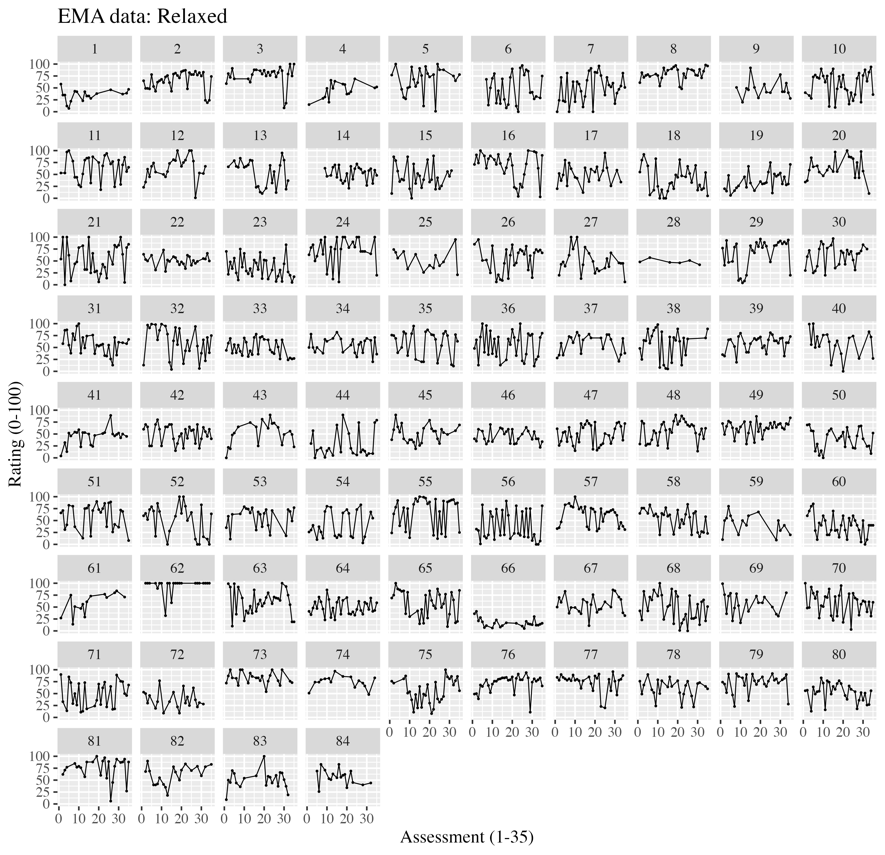


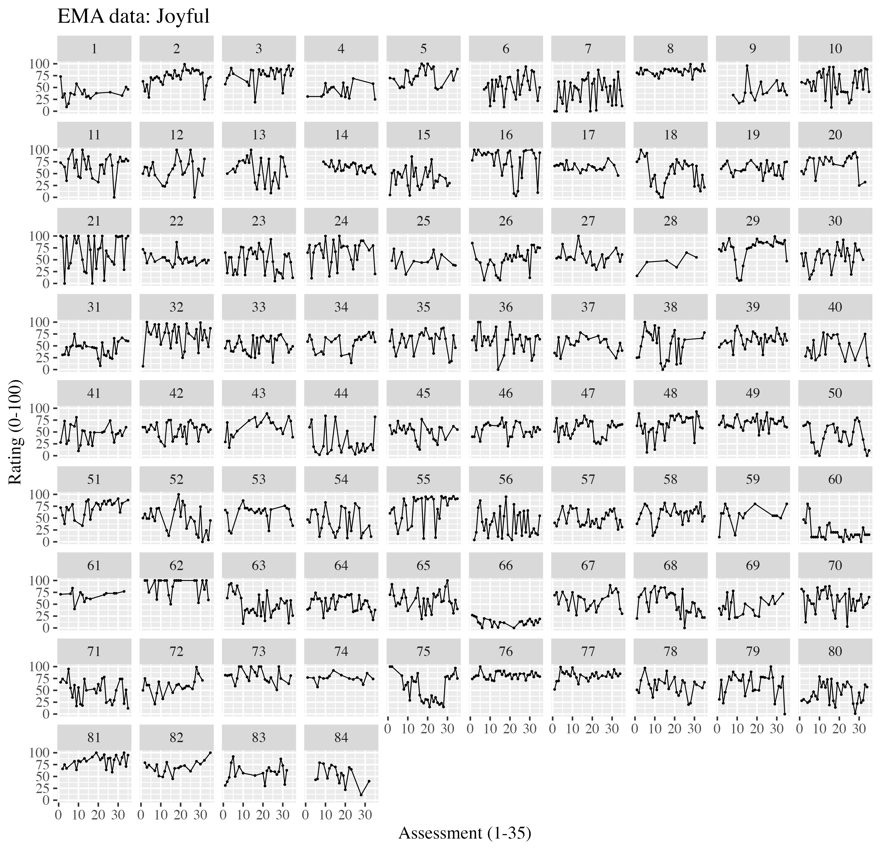


1. **Interrater reliability of qualitative data:**

A_topic = Ratings from two raters in their capacity as first rater

B_topic = Ratings from corresponding second rater

In the cases of multiple codings for one response, the one that was identical to the one of the other rater was used in SPSS.

The one missing coding was replaced by a 0 (automatically making it a non-match).

Inter-rater reliability for each open question was assessed separately:

- Ethica experience: “Can you describe your experience with the Ethica app? What did you like or dislike about using the app?”

The average measure intraclass correlation coefficient (ICC) was .615 with a 95% confidence interval from -.321 to .900 (*F*(9,9) = 2.768, *p* = .073).

- Frequency of EMA: “How did you feel about the amount of questionnaires in a day?”

The average measure intraclass correlation coefficient (ICC) was 1.

- Timing of EMA: “How was your experience with the timing of the questions? Time of day here means the moments in the day when you received the questions.”

The average measure intraclass correlation coefficient (ICC) was 1.

- Positive evaluation: “What did you like about participating in this study?”

The average measure intraclass correlation coefficient (ICC) was 1.

- Negative evaluation: “What did you not like about participating in this study?”

The average measure intraclass correlation coefficient (ICC) was 1.

- Measurement Reactivity: “What effect did answering the questions have on your daily life? We are curious about the positive and negative effects. If possible, please also explain which question(s) affected your daily life.”

The average measure intraclass correlation coefficient (ICC) was .996 with a 95% confidence interval from .986 to .999 (*F*(9,9) = 276.56, *p* < .001).

- Perceived measurement: “What do you think was measured by the daily questions?”

The average measure intraclass correlation coefficient (ICC) was 1.

- Participant suggestions: “Now that you know what we want to use the daily questions for, what would you change about the daily questions if you were on our research team?”

The average measure intraclass correlation coefficient (ICC) was 1.

1. **Missingness analysis**

EMA responses per participant per day; red line indicates 5 assessments per day.

**
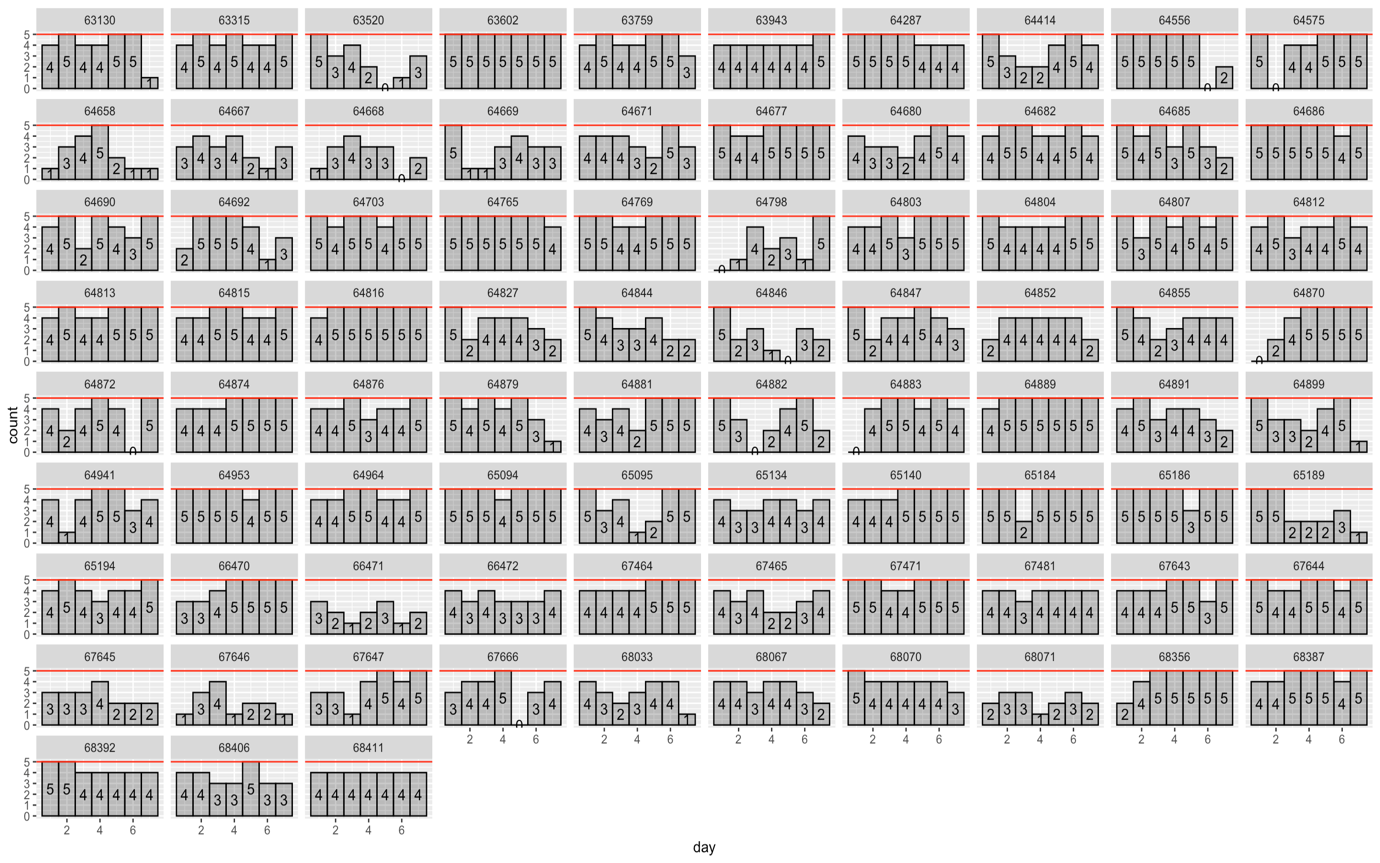
**

EMA responses per participant per assessment; red line indicated 7 days per assessment.

**
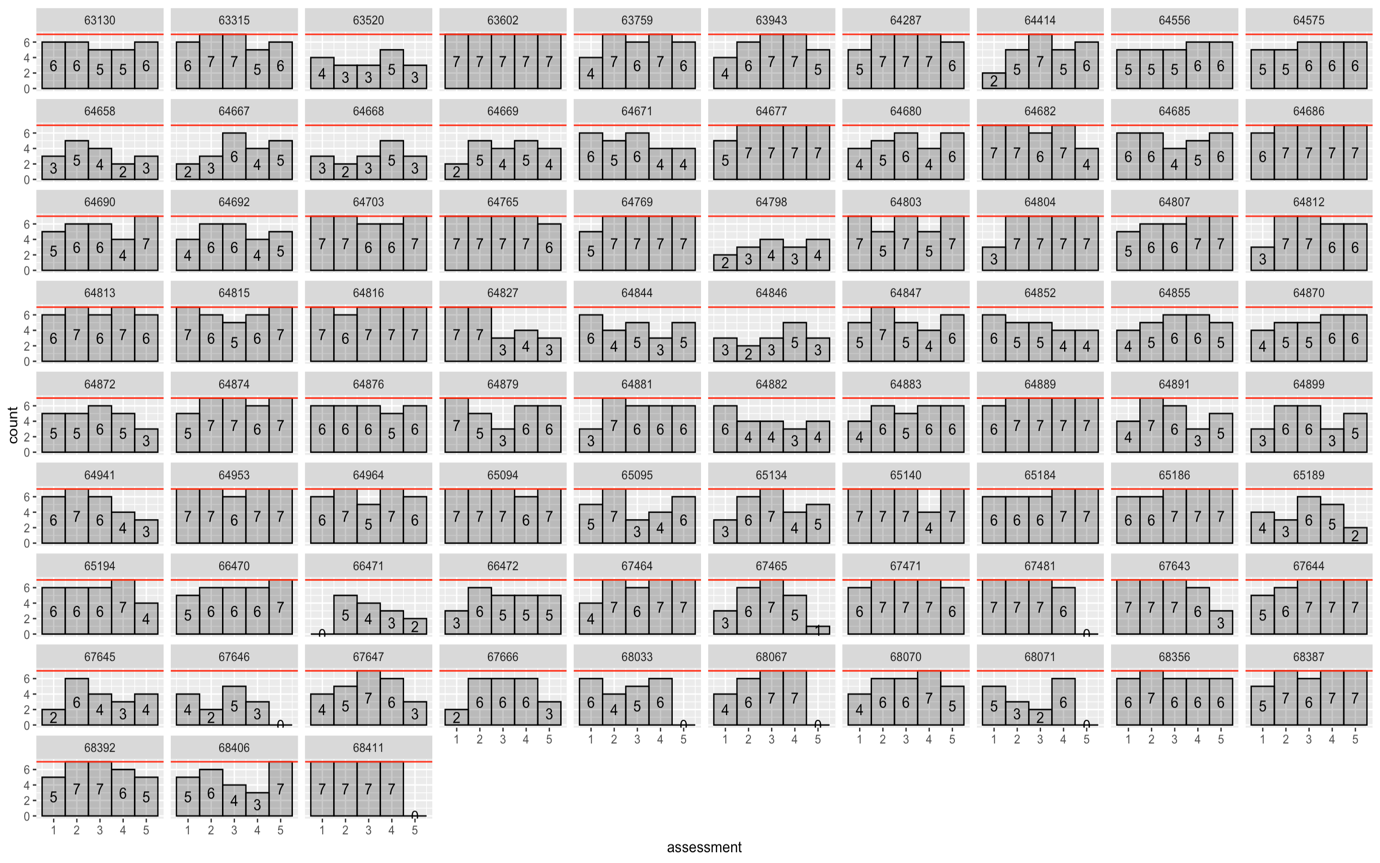
**

1. **Analysed qualitative data (Note: All data is translated from Dutch to English):**
2. Overall experience participating

Combined analysis of answers to questions “What did you like about participating in this study?” and “What did you not like about participating in this study?”

| Valence | Code | Explanation | Example | N | % |
| --- | --- | --- | --- | --- | --- |
| Positive | Insight into their own feelings_positive | Enjoying the reflection/insight they got into their own feelings through the day, in relation to smoking and/or generally | I thought it was cool to have more insight in how I feel when I do or don't smoke | 31 | 38,75 |
| Negative | Timing of the questions/time given | Disliked the timing of the questions and that they couldn't be answered after a while | If I didn't have time to open the app, I couldn't answer the questions anymore. | 31 | 38,75 |
| Negative | Neutral |  | nothing | 18 | 22,5 |
| Negative | Disliked the questions | Didn't like the questions | the same questions everytime again and again | 17 | 21,25 |
| Positive | Monitoring behaviour_positive | Enjoying monitoring own behaviour | Keeping track of when I smoke | 16 | 20 |
| Positive | Easy to do | The questions were easy and short | easy and fast | 14 | 17,5 |
| Positive | Liked the questions | Liked the questions | The short questions | 12 | 15 |
| Negative | insight into own feelings_ negative | Found it confronting, disliked the reflection they got into own feelings | It was confronting to see how much i smoked | 9 | 11,25 |
| Positive | Interesting to do | interesting to answer the questions | It was very interesting | 6 | 7,5 |
| Positive | Helping others/contributing to research | Like to help others and contribute to research this way | To help with the gathering of information | 5 | 6,25 |
| Positive | Missing response | There was no answer |  | 5 | 6,25 |
| Negative | missing response | There was no answer |  | 5 | 6,25 |
| Positive | Neutral |  | It was fine to do, to fill in a few questions per day doesn't hurt anybody. | 3 | 3,75 |
| Positive | Frequently being notified to answer questions | Like the amount of times there was a notification to answer the questions | I liked the questions on different times of the day | 2 | 2,5 |
| Positive | something to do | It was something to do in the day | That I had something every day to spend time on. | 2 | 2,5 |
| Positive | Motivation to change | The questions gave them motivation to change their smoking behaviour | That it was simple and motivated me to change | 1 | 1,25 |
| Positive | The app_positive | Liked using the app itself | The use of the app | 1 | 1,25 |
| Positive | Nothing | Didn't like anything | nothing | 1 | 1,25 |
| Negative | Reverse effect | The app caused them to want to smoke more | Sometimes the questions gave me an even bigger urge to smoke a cigarette. | 1 | 1,25 |
| Negative | The app_negative | Didn't like the app in general | The app | 1 | 1,25 |
| Negative | Stress | The questions caused stress | That I had stress sometimes to fill it in | 1 | 1,25 |

1. Experience with EMA application Ethica Data

Analysis of answers to the question: “Can you describe your experience with the Ethica app? What did you like or dislike about using the app?”

| Code | Explanation | Example | Count | % |
| --- | --- | --- | --- | --- |
| No problems | There were no problems during the installation or use of the Ethica app |  | 71 | 88,75 |
| Understanding of the app_positive | Finding the app easy to understand and use | I was quite positive about it because it was easy to understand | 41 | 51,25 |
| Functionality of the app_positive | The app functioned /worked well | Works perfectly | 13 | 16,25 |
| Missing answer | No answer was filled in |  | 15 | 18,75 |
| Overall positive_unclear reason | Positive about the app, but without giving a single reason | Good app | 11 | 13,75 |
| Notifications_positive | Positive about the notification system of the app | The app was nice to work with. You only have to log in once. It was also nice that you got a reminder if you only had 15 minutes left for your questionnaire | 7 | 8,75 |
| Notifications_negative | Negative about the notification system of the app | The app regularly dropped out, notifications that I had to turn on a certain way caused my battery to drain faster | 2 | 2,5 |
| Functionality of the app_negative | The app functioned/ didn't work well | System sometimes did not run smoothly | 2 | 2,5 |
| Understanding of the app_negative | Finding the app unclear and hard to use | The app was unclear | 1 | 1,25 |
| Ethica Experience_Data saving | The ethica experience was negative due to data not being saved well | The app was fine, but in the middle of the survey, my data was suddenly deleted so I had to start over. That was kind of annoying. | 2 | 2,5 |
| Difficulty answering | Questions were asked that the participant did not know the answer to | Many things were asked like number and is and I didn't know them | 1 | 1,25 |

1. Frequency and Timing of the EMA
   Combined analysis of answers to questions “How did you feel about the amount of questionnaires in a day?” and “How was your experience with the timing of the questions? Time of day here means the moments in the day when you received the questions.”

| Code | Explanation | Exmple | Count | % |
| --- | --- | --- | --- | --- |
| Amount_positive | Positive about the amount of questions | Doable | 54 | 67,5 |
| Satisfied with the timing | Is at least somewhat positive about (certain aspects of) the timing | Well, worked out for me, and we had plenty of room to respond | 43 | 53,75 |
| Bad timing_busy | Negative about the timing due to being busy | Sometimes I could not respond because of the timing | 23 | 28,75 |
| Too many questions | There were a lot of questions | maybe a bit much, but I understand the need for that | 22 | 27,5 |
| Missing answer | Answer is missing |  | 12 | 15 |
| Morning question_Too early | The questions in the morning were too early, usually due to being asleep. | In the morning I was sometimes still not awake | 10 | 12,5 |
| Dissatisfied_Lacks reason | Negative about the timing of the questionnaires, but no explanation why | Inconvenient | 4 | 5 |
| Evening questions_Too late | The questions in the evening were too late | Sometimes too late | 2 | 2,5 |
| Amount_Timing problem | Timing of the questions was problematic for the amount | Fine just a pity you only got an hour because sometimes I worked or had school | 1 | 1,25 |
| Unknown time | It was unclear at what specific times questionnaires had to be filled in | I did find it annoying what the you didn't know exactly what time they were coming. | 1 | 1,25 |
| Question type_negative | Other questions would have been better | Fine, just other questions would be better | 1 | 1,25 |
| Missed questions_Lacks reason | Missed questionnaires, but no mentions of being busy or not being able to answer the questions | Fine though, only the first one I missed at times | 1 | 1,25 |
| Dissatisfied_Afternoon | Negative about the afternoon timing | In the afternoon, it was a bit harder to answer quickly | 1 | 1,25 |
| Forgetting | Forgot to fill in the questionnaire | Fine. Only at night I quickly forget | 1 | 1,25 |

1. Measurement reactivity

Analysis of answers tot he question “What effect did answering the questions have on your daily life? We are curious about the positive and negative effects. If possible, please also explain which question(s) affected your daily life.” Only participants who reported measurement reactivity greater than “not at all” were shown this question, N = 62.

| Code | Explanation | Example | Count | % |
| --- | --- | --- | --- | --- |
| awareness | The questions gave an insight into own feelings/created awareness of smoking behaviour | I was more conscious of how much I smoked in a day | 37 | 46,25 |
| Not much/no effect | The effect was small to none existing | next to nothing | 23 | 28,75 |
| Less smoking/positive effect | The questions caused less smoking behaviour, it had a positive effect | I smoke less | 12 | 15 |
| Negative feelings | The questions caused negative feelings, it was confronting | More often sad than normally | 8 | 10 |
| missing response | no answer was given |  | 6 | 7,5 |
| Structure | The questions created structure in daily life | Answering the questions had a pretty positive effect, I had the feeling that I had more structure in my head because of the questions | 1 | 1,25 |
| Took some time | The questions took some time out of the day | Had to take some time for it sometimes | 1 | 1,25 |
| remember | Questions demanded that smoking behaviour is remembered | Remembering how much I smoke | 1 | 1,25 |
| increased alertness/phone use | The questions caused an increased alertness/ more use of phone | I kept checking my phone to not miss it | 1 | 1,25 |

1. Perceived measurement

Analysis of answers to the question ““What do you think was measured by the daily questions?”

| Code | Explanation | Example | Count | % |
| --- | --- | --- | --- | --- |
| Emotions/mood | Thought that the questions were about measuring emotions/mood | Your emotion, and the influence of smoking on your mental health. | 43 | 53,75 |
| smoking behaviour | Thought that questions were about smoking behaviour | Average cigarettes in certain age groups | 40 | 50 |
| Missing response | no answer was given |  | 6 | 7,5 |
| Daily routine | Thought that questions were about daily routine | Daily routine of a smoker. | 4 | 5 |
| Neutral | Didn't think anything, were neutral | .' Or 'x' or 'good' | 4 | 5 |
| unclear | Didn't know what it was about | That was a little unclear | 3 | 3,75 |

1. Participant suggestions

Analysis of answers to the question “Now that you know what we want to use the daily questions for, what would you change about the daily questions if you were on our research team?”

| Code | Explanation | Example | Count | % |
| --- | --- | --- | --- | --- |
| Nothing | Everything looked fine, change nothing | nothing | 43 | 53,75 |
| Suggestions of questions | Suggestion of a new question/another idea | The question: if you look back, did you really crave that cigaret? | 22 | 27,5 |
| Missing response | No answer was given to this question |  | 7 | 8,75 |
| Other formulation of question | Suggest another formulation of a question/change the existing questions | The question: I have thought about smoking a cigaret in the last hour. Change this with: I crave a cigaret. | 6 | 7,5 |
| Timing of the questions | Change the timing of the questions/how long the questions pop up | Since youth stay up late, I would make sure the questions come at a later time as well, maybe even in the night. | 2 | 2,5 |
| The app | Change the app itself | A more attractive app | 1 | 1,25 |
| Unclear | It was unclear, don't know what to change | I don't know, I think I need more information about how the app is supposed to look in the end. | 1 | 1,25 |

List of participants’ suggestions:

- I would focus more on quitting and not on emotions
- Maybo go deeper, for example if you get the question: are you restless at this moment? And somebody says that they are very restless, ask further why
- Why did you smoke? Often you smoke because of a feeling or impulse or to suppress something. I got addicted because of traumatic things I've been through. If you can recognize the feelings that arise before someone smokes, you can prevent them from smoking again. If I couldn't do this, I would've smoked a lot more.
- Maybe what you smoke?
- Go deeper into the effects of smoking instead of general questions
- More variety, so you dwell on it and think about it.
- Maybe a question like: how much do you want to stop smoking at this time/ are you planning on smoking?
- I would ask what encourages youth to smoke or why they do it on certain times
- Maybe sadness and happy together in a question like relaxed and restless? I don't know exactly which ones, but just do you feel more relaxed or restless? Because they are pretty much opposites.
- Maybe something with peer pressure
- Maybe a choice of different moods on which you can elaborate
- maybe about concentrating was unclear
- Maybe ask another reason why someone wants to smoke
- Maybe ask why the urge to smoke
- I would ask firstly if you smoked today and then the other questions
- Also, involve the influence of situations happening in life in the questions which ask about feelings. There is not always a direct link between smoking behavior and the feeling of the person
- Also ask for location/occassion. I noticed when the questions came and I was outside, the urge to smoke was higher than when I was inside.
- The question: If you look back, did you really crave that cigarette?
- More questions
- Not how much after your last answer, but the total per day
- Do you have stress or has someone been mad?
- Maybe the question: 'did you last smoke because of stress or other bad moods?' I understand if the question is to direct
- What was the reason to smoke
- I would change the questions a bit more, so there's more variety
- I would definitely make a question about smoking alone or with people, in this way you can make the connection that smoking with people causes a rise in smoking behavior (if that is the case ofcourse)
- The question: I thought about smoking the past hour, change it with: I fhave the urge to smoke a cigarette
- More specific questions

1. **Exploratory analyses**

Since participants rated measurement reactivity as moderate, with some thinking they smoked less during the EMA week and others reporting more insight into their smoking, we conducted exploratory analyses to test any changes in daily smoking over the 7 days of EMA. First, we tested the relationship between daily smoking and day of EMA (1 to 7) in the whole sample using a multilevel model with data nested in participants. We accounted for random intercepts and used restricted maximum likelihood estimation. Day of EMA did not predict daily smoking, *B* = -0.16, *t*(503) = -1.84, *SE* = 0.09, *p* = .067. Second, we tested the same model in a subset of participants who reported experiencing an impact of the EMA in their daily life as greater than or equal to 3 out of 7 (*n* = 48). Again, day of EMA did not predict smoking, *B* = -0.15, *t*(294) = -1.93, *SE* = 0.08, *p* = .055.

The correlation between the retrospective positive affect scores and mean EMA scores on positive affect was 0.3, lower than expected. Therefore, we also tested how each of the EMA positive affect items correlated with the retrospective positive affect score. Mean scores on the “joyful” item correlated moderately with retrospective positive affect (*r* = .46, *p* < .001) and mean scores on the “relaxed” item correlated weak-moderately with retrospective positive affect (*r* = .34, *p* = .002).

We also plotted missingness data per day (days 1-7 of EMA) and per assessment (assessments 1-5) across the sample (*Figure 2 in manuscript*) and per participant (*Supplementary Materials V*). Missingness did not seem to vary based on assessment time or day. Further, we tested the relationship between demographic variables such as gender, *t*(54) = 1.32; *p* = .191, age, *R^2^* = -0.01, *F*(1, 82) = -0.70, *p* = .580, weekly smoking at pre-test, *R^2^* = -0.01, *F*(1, 82) = 0.03, *p* = .445, and education level, *t*(76.2) = 0.68; *p* = .502, did not predict compliance rates.

References

Cox, L.S., Tiffany, S.T., Christen, A.G., 2001. Evaluation of the brief questionnaire of smoking urges (QSU-brief) in laboratory and clinical settings. Nicotine Tob. Res. 3, 7–16. https://doi.org/10.1080/14622200124218

Heatherton, T.F., Kozlowski, L.T., Frecker, R.C., Fagerström, K.O., 1991. The Fagerström Test for Nicotine Dependence: a revision of the Fagerström Tolerance Questionnaire. Br. J. Addict. 86, 1119–1127. https://doi.org/10.1111/j.1360-0443.1991.tb01879.x

Littel, M., Franken, I., Muris, P., 2011. Psychometric properties of the brief Questionnaire on Smoking Urges (QSU-Brief) in a Dutch smoker population. Neth. J. Psychol. 1–20.

Peeters, F.P.M.L., Ponds, R.H.W.M., Vermeeren, M.T.G., 2004. Affectivity and self-report of depression and anxiety. Tijdschr. Voor Psychiatr. 240–250.

Scholten, H., Luijten, M., Granic, I., 2019. A randomized controlled trial to test the effectiveness of a peer-based social mobile game intervention to reduce smoking in youth. Dev. Psychopathol. 31, 1923–1943. https://doi.org/10.1017/S0954579419001378

Smith, S.S., Piper, M.E., Bolt, D.M., Kaye, J.T., Fiore, M.C., Baker, T.B., 2021. Revision of the Wisconsin Smoking Withdrawal Scale: Development of Brief and Long Forms. Psychol. Assess. 33, 255–266. https://doi.org/10.1037/pas0000978

Tiffany, S.T., Drobes, D.J., 1991. The development and initial validation of a questionnaire on smoking urges. Br. J. Addict. 86, 1467–1476. https://doi.org/10.1111/j.1360-0443.1991.tb01732.x

Vink, J.M., Willemsen, G., Beem, A.L., Boomsma, D.I., 2005. The Fagerström Test for Nicotine Dependence in a Dutch sample of daily smokers and ex-smokers. Addict. Behav. 30, 575–579. https://doi.org/10.1016/j.addbeh.2004.05.023

Watson, D., Clark, L.A., Tellegen, A., 1988. Development and validation of brief measures of positive and negative affect: the PANAS scales. J. Pers. Soc. Psychol. 54, 1063–1070. https://doi.org/10.1037//0022-3514.54.6.1063
